# Preclinical Evaluation of HER2-Targeting DARPin G3: Impact of Albumin-Binding Domain (ABD) Fusion

**DOI:** 10.3390/ijms25084246

**Published:** 2024-04-11

**Authors:** Sergey M. Deyev, Maryam Oroujeni, Javad Garousi, Torbjörn Gräslund, Ruonan Li, Alia Hani Binti Rosly, Anna Orlova, Elena Konovalova, Alexey Schulga, Anzhelika Vorobyeva, Vladimir Tolmachev

**Affiliations:** 1 Molecular Immunology Laboratory, Shemyakin & Ovchinnikov Institute of Bioorganic Chemistry, Russian Academy of Sciences, 117997 Moscow, Russia; biomem@mail.ru (S.M.D.); elena.ko.mail@gmail.com (E.K.); schulga@gmail.com (A.S.); 2 Research Centrum for Oncotheranostics, Research School of Chemistry and Applied Biomedical Sciences, Tomsk Polytechnic University, 634050 Tomsk, Russia; 3 Department of Immunology, Genetics and Pathology, Uppsala University, 751 85 Uppsala, Sweden; maryam.oroujeni@igp.uu.se (M.O.); garousi@kth.se (J.G.); alia-hani-binti.rosly.1042@student.uu.se (A.H.B.R.); anzhelika.vorobyeva@igp.uu.se (A.V.); 4 Affibody AB, 171 65 Solna, Sweden; 5 Department of Protein Science, KTH—Royal Institute of Technology, 106 91 Stockholm, Sweden; torbjorn@kth.se (T.G.); ruonan@kth.se (R.L.); 6 Department of Medicinal Chemistry, Uppsala University, 751 83 Uppsala, Sweden; anna.orlova@ilk.uu.se

**Keywords:** DARPin G3, albumin-binding domain (ABD), HER2, SKOV-3 xenograft, Lutetium-177 (^177^Lu), SPECT imaging

## Abstract

Designed ankyrin repeat protein (DARPin) G3 is an engineered scaffold protein. This small (14.5 kDa) targeting protein binds with high affinity to human epidermal growth factor receptor 2 (HER2). HER2 is overexpressed in several cancers. The use of the DARPin G3 for radionuclide therapy is complicated by its high renal reabsorption after clearance via the glomeruli. We tested the hypothesis that a fusion of the DARPin G3 with an albumin-binding domain (ABD) would prevent rapid renal excretion and high renal reabsorption resulting in better tumour targeting. Two fusion proteins were produced, one with the ABD at the C-terminus (G3-ABD) and another at the N-terminus (ABD-G3). Both variants were labelled with ^177^Lu. The binding properties of the novel constructs were evaluated in vitro and their biodistribution was compared in mice with implanted human HER2-expressing tumours. Fusion with the ABD increased the retention time of both constructs in blood compared with the non-ABD-fused control. The effect of fusion with the ABD depended strongly on the order of the domains in the constructs, resulting in appreciably better targeting properties of [^177^Lu]Lu-G3-ABD. Our data suggest that the order of domains is critical for the design of targeting constructs based on scaffold proteins.

## 1. Introduction

The human epidermal growth factor receptor type 2 (HER2) is one of the transmembrane receptor tyrosine kinases belonging to the epidermal growth factor receptor (EGFR) family. HER2 signalling is triggered by its homodimerisation or heterodimerisation with other members of the HER family, which causes phosphorylation of tyrosine residues of the receptor’s cytoplasmic domain. The phosphorylation initiates a range of downstream signalling cascades, which regulate cell proliferation, apoptosis, and motility. The overexpression of HER2 has been documented in breast, gastric/gastroesophageal, and ovarian cancers [1]. In addition, overexpression of HER2 receptors has been observed in other malignancies such as endometrial, lung, and head and neck cancers [2]. The overexpression of HER2 results in such features of malignancy as a high rate of cell proliferation, an increased migration capability, and apoptosis suppression [3]. Several HER2-targeting monoclonal antibody (mAb)-based therapeutics have demonstrated remarkable efficacy in the treatment of breast and gastroesophageal cancers, increasing the survival of the patients [4]. Unfortunately, the development of resistance to such therapies is almost inevitably observed after some time.

MAbs, labelled with cytotoxic radionuclides, such as beta- or alpha-particle emitters, have been evaluated as therapeutic agents in radioimmunotherapy [5]. The use of radionuclides has an advantageous cross-fire effect when emitted particles damage the DNA of not only targeted cells but also of neighbour malignant cells. This might help to overcome a possible heterogeneity in target expression. In addition, radionuclides do not act as substrates for efflux pumps, which minimises the risk of resistance development. However, radiolabelled antibodies accumulate slowly in tumours and mainly around blood vessels. A protracted residence of antibodies in circulation causes irradiation of the bone marrow, which is very radiosensitive [6]. Thus, the delivery of a sterilising dose to tumours using radiolabelled antibodies is not feasible without high doses to critical normal tissues. As major issues with radioimmunotherapy are associated with the large size of the mAbs, the development of smaller alternatives would be attractive.

Appreciable efforts have been invested in developing targeting agents, which are smaller than immunoglobulins. To develop radionuclide imaging probes, several engineered scaffold proteins (ESPs), namely, affibody molecules, albumin-binding domain-derived affinity proteins (ADAPTs), and designed ankyrin repeat proteins (DARPins) [7,8,9,10,11] have been tested. The use of these small (molecular weight 6–18 kDa) targeting probes offers several advantages compared to the bigger (150 kDa) mAbs. Preclinical studies have demonstrated that ESPs can bind specifically to their molecular targets in vivo [8], and have a high rate of accumulation in tumours. Furthermore, they clear promptly from blood, providing an excellent imaging contrast [8]. Moreover, initial clinical evaluation has demonstrated [12] that ESP-based agents can visualise HER2-expressing tumours with high contrast, and their injection is well tolerated and safe.

One of the promising classes of ESPs is the designed ankyrin repeat proteins. The DARPin scaffold is built from four or five amino acid repeats containing two antiparallel α-helices and a β-turn. DARPins are water soluble and stable at elevated temperatures. DARPins can be produced in prokaryotes, which makes their manufacturing cheaper than the production of antibodies. High-affinity DARPins binding to different molecular structures have been developed for several applications including tumour targeting [10]. Like other ESPs, DARPins have a short biologic half-life in blood. They pass through the glomerular membrane because of their small size, which is lower than the renal filtration threshold (60 kDa). The DARPin G3 (14–15 kDa) was developed via an affinity maturation procedure and showed a high affinity (K_D_ = 90 pM) to the HER2 receptor [13,14]. A Phase I clinical study of the ^99m^Tc-labeled G3 DARPin demonstrated that this tracer can visualise HER2 expression in breast cancer 2–4 h after injection [12]. However, the highest radioactivity uptake was detected in the kidney, where DARPins are reabsorbed in the proximal tubules, followed by rapid internalisation. As a result, the high accumulation of activity in the kidney prevents the direct use of G3 for targeted radionuclide therapy. Using pharmacological approaches to prevent the kidney uptake of the radiolabelled DARPins has resulted in only 2–2.5 fold reduction, which is insufficient for radionuclide therapy [15].

A number of strategies to improve the pharmacokinetics, extend blood residence and reduce renal uptake of small targeting agents, like ESPs is reviewed by Kontermann [16]. One strategy is to increase the protein size to above the renal filtration cut-off. Chemical modification by conjugation with polyethylene glycol (PEGylation) of DARPins has been described previously [8]. In addition, fusion to albumin-binding DARPins [17], binding to unstructured protein modules like PAS (polypeptide containing proline, alanine, and serine) [18,19] or XTEN (polypeptide containing proline, alanine, serine, glycine and glutamate) [19] have been investigated. An alternative strategy is the use of labelling chemistry that provides non-residualising radiocatabolites, such as radioiodination [14,20]. Such a strategy enables a decrease in the retention of activity in the kidney by several fold and improves the tumour-to-kidney ratio.

Among the strategies mentioned above, the fusion of targeting protein (i.e., an affibody molecule) with an albumin-binding domain (ABD) is an appealing strategy for half-life extension [21]. Albumin is the most abundant protein in the plasma, with a long circulatory half-life of 19 days. A fusion with the ABD enables the binding to serum albumin following administration, raising the total molecular weight of the ABD-fused ESP-albumin adduct to above the renal cut-off. As a result, this fusion prevents rapid glomerular filtration and tubular reabsorption, and the half-life of the targeting protein in the blood is prolonged, which could lead to more efficient delivery into tumours [22]. On top of that, the efficiency of the receptor-mediated recycling pathway involving the neonatal Fc receptor (FcRn) contributes to a further extension of the circulatory half-life of ABD-fused ESPs [23,24]. Importantly, the molecular weight of the ESP-ABD-albumin adduct is below 90 kDa, which is smaller than the molecular weight of an IgG antibody. Thus, such constructs should have more efficient extravasation and diffusion compared to an IgG.

**Figure 1:**
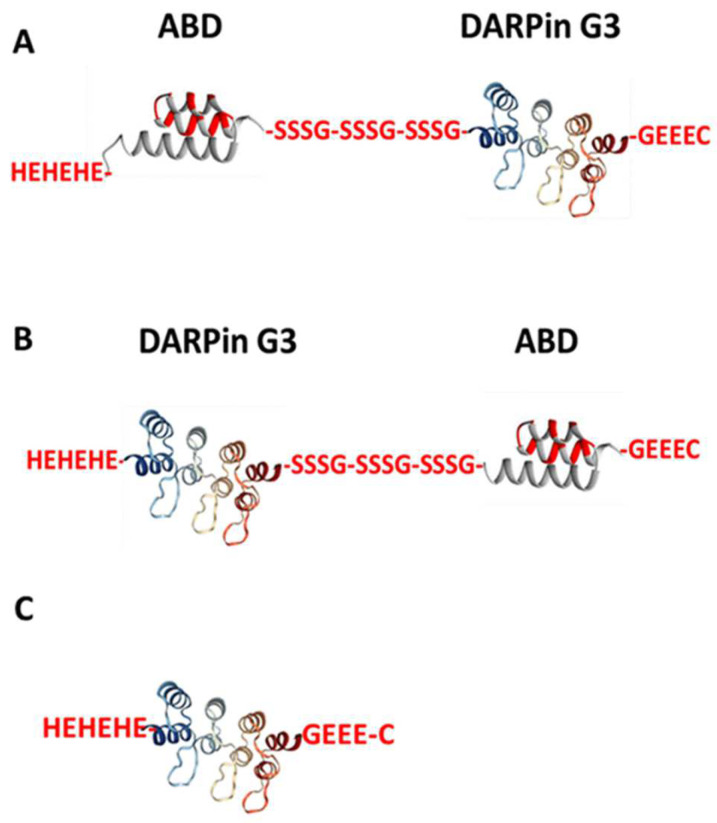
Schematic structures of (**A**) ABD-G3, (**B**) G3-ABD, and (**C**) non-ABD-fused DARPin G3 (a control protein). All variants were coupled with DOTA chelator at C-terminus for labelling with ^177^Lu.

Previous studies have demonstrated that the fusion of an ABD to affibody molecules and ADAPT6 can be used to efficiently target human cancer xenografts with high HER2 expression in mice [25,26]. These studies have shown an increased tumour uptake compared with the non-fused ESP, while the uptake in the kidney was appreciably reduced. We hypothesised that a fusion to ABD would also enhance the residence of DARPins in blood, reduce their renal uptake and increase their tumour uptake. To test this hypothesis, we created two fusion proteins (Figure 1). In the variant designated ABD-G3, the ABD was positioned at the N-terminus of the DARPin (Figure 1A). In the variant G3-ABD, the ABD was placed at the C-terminus (Figure 1B). The domains were connected using hydrophilic (S_3_G)_3_-linkers, which demonstrated a capacity to prevent mutual steric hindrance in homo- and heterodimeric constructs based on ADAPT6 and the ABD [27]. The N-termini of both constructs were fitted with (HE)_3_-tags, which enables immobilised metal ion affinity chromatography (IMAC) purification and improves the biodistribution of ESPs [28]. A unique cysteine was introduced at the C-terminus of each construct for site-specific conjugation of a maleimido derivative of the 1, 4, 7, 10-tetraazacyclododecane-1, 4, 7, 10-tetracetic acid (DOTA) chelator, which provides a stable coupling of ^177^Lu [29]. ^177^Lu is most commonly used clinically for targeted radionuclide therapy because of its favourable decay scheme [30].

As a non-ABD-fused control, a G3 variant containing a (HE)_3_-tag and a GEEEC moiety at the N- and C-termini, respectively, was designed. This construct was used in a biodistribution experiment to evaluate the effect of G3 fusion with the ABD on biodistribution properties. The study plan included the production of the constructs, coupling with the DOTA chelator, labelling with the radionuclide ^177^Lu and evaluation of their binding specificity, affinity, and cellular processing in vitro, and the measurement of their biodistribution in vivo. 

## 2. Results

### 2.1. Production, Purification, and Characterisation of the ABD-Fused DARPin G3 Variants and G3 and Conjugation of the DOTA Chelator

The constructs were produced according to specifications and successfully coupled with DOTA. According to the liquid chromatography–mass spectrometry (LC-MS) analysis (Figure S2), the use of an eleven-fold molar excess of DOTA maleimide for coupling to freshly reduced DARPin provided full conjugation of the chelator to all proteins. No unconjugated DARPins were found. The agreement between the calculated and the found molecular masses of the proteins was within 1 Da, i.e., within the accuracy of the method. Amino acid sequences of the DARPin constructs are shown in Figure S1.

The surface plasmon resonance measurements (Table 1, Figure S3) showed that both G3-ABD-DOTA and ABD-G3-DOTA have strong binding to albumin. The binding to human serum albumin was stronger than the binding to the murine counterpart in both cases. The binding of ABD-G3-DOTA to both human and murine albumin was approximately one order of magnitude weaker compared with G3-ABD-DOTA.

### 2.2. Radiolabelling of the Probes with 177Lu and In Vitro Stability

Radiolabelling of the anti-HER2 DARPins containing DOTA as a chelator with ^177^Lu was performed at 60 °C. The radiochemical yield was 22 ± 9% and 88 ± 9% for [^177^Lu]Lu-G3-ABD and [^177^Lu]Lu-ABD-G3, respectively. The radiochemical purity of the radiolabelled conjugates after purification using NAP-5 columns was >98% according to iTLC measurements. Radio-high-performance liquid chromatography (HPLC) chromatograms of the purified conjugates (Figure S4) were in excellent agreement with the iTLC data. The specific activity was 1 MBq/µg. The release of ^177^Lu from the purified constructs was less than 2% after 1 h incubation at 37 ^o^ C in the presence of a 500-fold molar excess of EDTA. After incubation in murine serum at 37 °C for 48 h, the protein-associated activity was 96.2 ± 1.0% and 87.3 ± 3.0% for [^177^Lu]Lu-ABD-G3 and [^177^Lu]Lu-G3-ABD, respectively.

Radiolabelling of the non-ABD-fused G3 was performed in the same way as labelling of G3-ABD, resulting in a radiochemical purity over 99 ± 1%. Labelling of Z_HER2:2891_-ABD with ^177^Lu was performed according to previously published protocols [31] providing a radiochemical purity of over 98%. After incubation in murine serum at 37 °C for 48 h, 93.6 ± 5.1% activity was associated with [^177^Lu]Lu-G3.

### 2.3. In Vitro Studies

**Figure 2:**
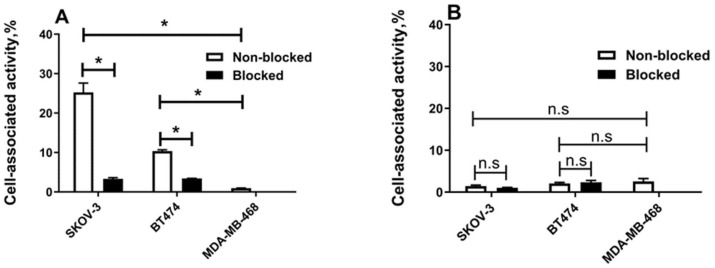
Specificity of [^177^Lu]Lu-labelled G3 binding in vitro to tumour cell lines without the addition of albumin. (**A**) [^177^Lu]Lu-G3-ABD and (**B**) [^177^Lu]Lu-ABD-G3 binding to SKOV-3 and BT-474 (HER2-positive) and MDA-MB-468 (HER2-negative) cell lines. For the pre-saturation of HER2 receptors, an excess of a non-radioactive DARPin G3 was added before adding the labelled conjugate. ANOVA test (with Bonferroni’s correction for multiple comparisons) was performed to test if the difference was significant (*p* < 0.05). Asterisk (*) marks significant (*p* < 0.05) difference; n.s. that the difference is not significant.

**Figure 3:**
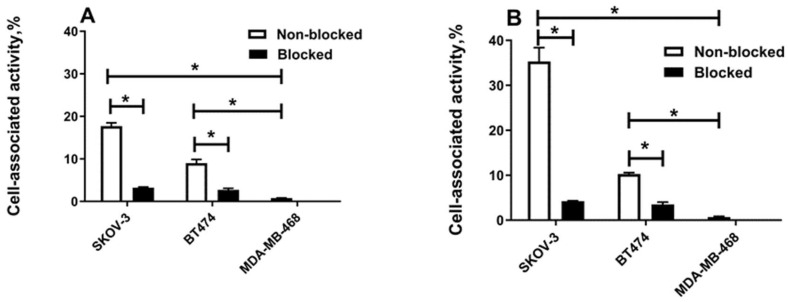
Specificity of [^177^Lu]Lu-labelled G3 binding in vitro to tumour cell lines in the presence of human serum albumin. (**A**) [^177^Lu]Lu-G3-ABD and (**B**) [^177^Lu]Lu -ABD-G3 binding in vitro to SKOV-3 and BT-474 (HER2-positive) and MDA-MB-468 (HER2-negative) cell lines in the presence of 100 nM human serum albumin. For the pre-saturation of HER2 receptors, an excess of a non-radioactive DARPin G3 was added before adding the labelled conjugate. ANOVA test (with Bonferroni’s correction for multiple comparisons) was performed to test if the difference was significant (*p* < 0.05). Asterisk (*) marks a significant (*p* < 0.05) difference.

The results of the in vitro binding assay revealed a strong impact of the protein’s architecture on specific binding to HER2-expressing cells SKOV-3 and BT-474 (Figure 2 and Figure 3). When the test was performed without adding human serum albumin, the binding of [^177^Lu]Lu-G3-ABD was significantly (*p* < 0.0005) decreased when the cells were pre-saturated with the non-labelled DARPin G3-ABD (Figure 2A), confirming the HER2-specific binding. Furthermore, [^177^Lu]Lu-G3-ABD binding to HER2-positive cell lines was significantly (*p* < 0.001) higher than binding to HER2-negative MDA-MB-486 cells. In vitro binding of [^177^Lu]Lu-G3, the non-ABD-fused control protein to these cell lines was also HER2-specific in these conditions (Figure S5). However, the binding of [^177^Lu]Lu-ABD-G3 to both cell lines was appreciably lower than the binding of [^177^Lu]Lu-G3-ABD, and could not be displaced by adding an excess of unlabelled protein. There was no difference between binding to HER2-positive and HER2-negative cells, i.e., the interaction was unspecific (Figure 2B).

The test outcome was different in the presence of human serum albumin (Figure 3). The cell-bound fraction of [^177^Lu]Lu-ABD-G3 increased compared with the cell-bound fraction in the absence of albumin. The binding of [^177^Lu]Lu-ABD-G3 to HER2-pistive cell lines was saturable and much higher than the binding to HER2-negative cells, i.e., it was specific. The specific binding of [^177^Lu]Lu-G3-ABD remained the same in these conditions.

The results of the InteractionMap analysis of the interactions (association and dissociation) of the ^177^Lu-labelled proteins with the HER2-expressing SKOV-3 cell line are presented in Table 2. The use of a Langmuir 1:2 model enabled the best fitting of the association and dissociation curves for [^177^Lu]Lu-G3-ABD. The affinity (apparent equilibrium dissociation constant) of the strongest interaction was approximately 1 nM for both ABD-fused constructs. The abundance of the strongest interaction for [^177^Lu]Lu-ABD-G3 (K_D1_ = 1290 ± 920 pM, % weight _1_ = 38) was lower than the binding of [^177^Lu]Lu-G3-ABD (K_D1_ = 901 ± 169 pM, %weight _1_ = 54). The affinity of [^177^Lu]Lu-G3 was similar to the affinities of [^177^Lu]Lu-ABD-G3 and [^177^Lu]Lu-G3-ABD in the presence of albumin.

**Figure 4:**
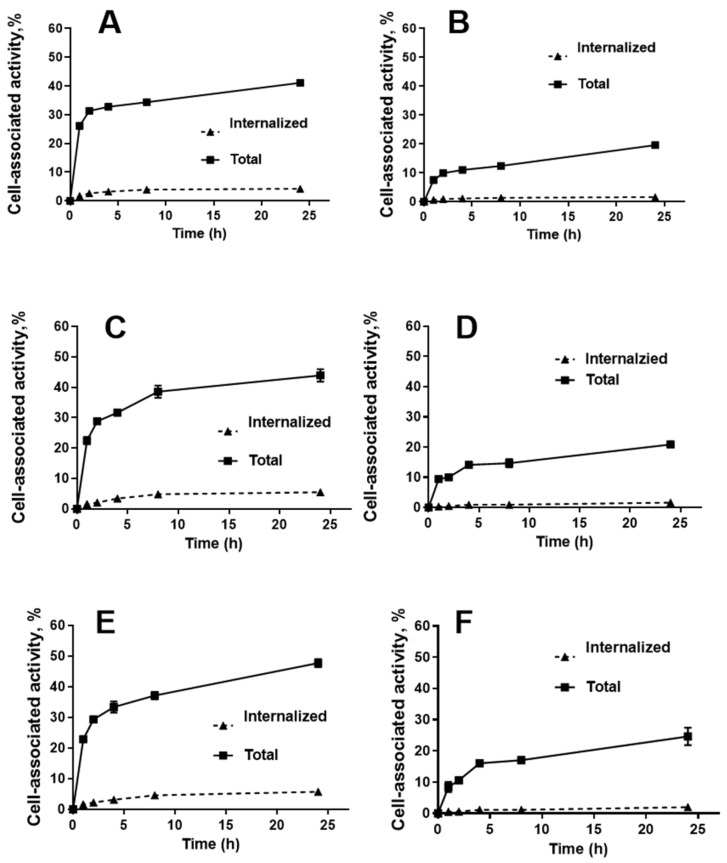
Cellular processing of [^177^Lu]Lu-labelled G3-ABD and ABD-G3. (**A**,**C**,**E**) Processing by SKOV-3 and (**B**,**D**,**F**) BT-474 cells. (**A**,**B**) show the binding and processing of [^177^Lu]Lu-G3-ABD without the addition of albumin. (**C**,**D**) show the binding and processing of [^177^Lu]Lu-G3-ABD with the addition of albumin. (**E**,**F**) show the binding and processing of [^177^Lu]Lu-ABD-G3 with the addition of albumin. The cells were incubated with 2 nM of the conjugates.

The cellular processing data (Figure 4) showed that the pattern of binding and internalisation in the presence of albumin was similar for [^177^Lu]Lu-G3-ABD and [^177^Lu]Lu-ABD-G3. The cell-associated activity increased rapidly during the first 2 h of incubation, but then the association rate decreased. The internalisation was relatively slow, reaching approximately 10% of the total cell-associated activity after 24 h incubation. The level of binding to SKOV-3 cells was higher than to BT-474 cells. The same pattern was observed for [^177^Lu]Lu-G3-ABD without adding albumin. Due to the lack of specific interaction at the test concentration, cellular processing of [^177^Lu]Lu-ABD-G3 was not evaluated without adding albumin.

### 2.4. In Vivo Studies

**Figure 5:**
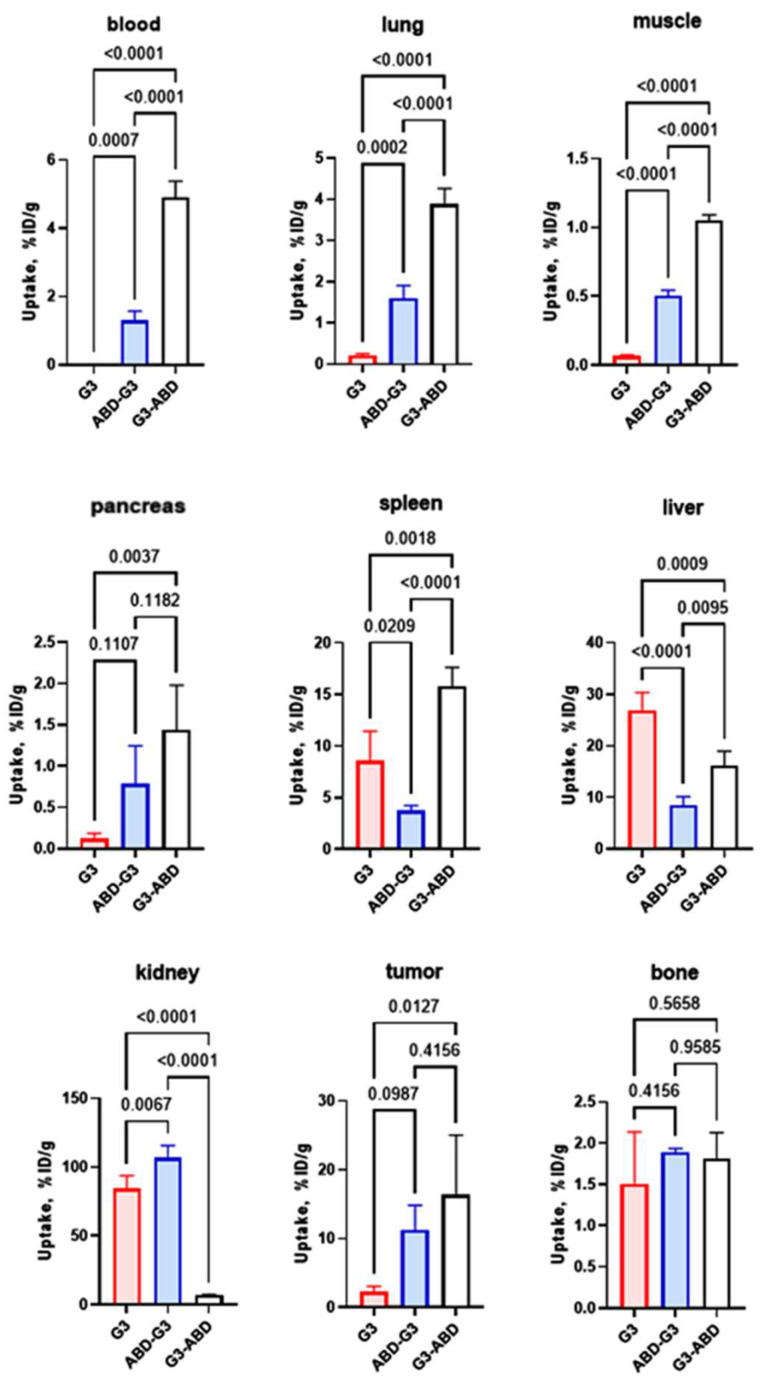
Effect of ABD fusion and the construct’s design on the biodistribution of ^177^Lu-labelled G3 derivatives in nude mice bearing SKOV-3 xenografts 48 h after injection. *p*-values were calculated using one-way ANOVA with Tuckey correction for multiple comparisons.

The effect of fusion with the ABD on the biodistribution of ^177^Lu-labeled G3 DARPin-based constructs 48 h after injection is shown in Figure 5. The fusion with the ABD resulted in significantly higher retention of both [^177^Lu]Lu-G3-ABD and [^177^Lu]Lu-ABD-G3 in blood compared with the control [^177^Lu]Lu-G3 (non-ABD-fused DARPin). Notably, the blood concentration of [^177^Lu]Lu-G3-ABD was approximately four-fold higher compared with the concentration of the variant where the ABD was fused with the N-terminus of G3. The renal uptake of [^177^Lu]Lu-G3-ABD was significantly (*p* < 0.05, one-way ANOVA) lower compared with the renal uptake of [^177^Lu]Lu-G3. However, the renal uptake of [^177^Lu]Lu-ABD-G3 was significantly (*p* < 0.05, one-way ANOVA) higher compared with the renal uptake of [^177^Lu]Lu-G3. The uptake of the constructs in the lung, muscle, and pancreas followed the pattern of the uptake in the blood. The pattern was different in the liver and spleen. The hepatic uptake of [^177^Lu]Lu-G3 was the highest. The uptake of this construct in the spleen was significantly (*p* < 0.05, one-way ANOVA) higher than the uptake of [^177^Lu]Lu-ABD-G3 but lower than the uptake of [^177^Lu]Lu-G3-ABD. The uptake of [^177^Lu]Lu-G3-ABD in HER2-expressing tumours was significantly (*p* < 0.05, one-way ANOVA) higher than the uptake of [^177^Lu]Lu-G3. [^177^Lu]Lu-ABD-G3 had a tendency of higher tumour uptake than [^177^Lu]Lu-G3, but the difference was not statistically significant. There was no significant difference in tumour uptake between [^177^Lu]Lu-G3-ABD and [^177^Lu]Lu-ABD-G3. Interestingly, the bone uptake at this time point was equal for all tested compounds.

**Figure 6:**
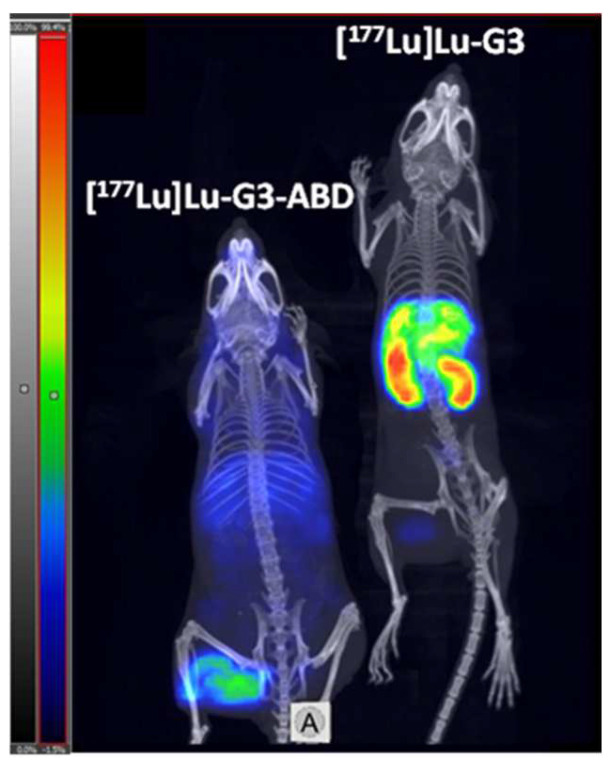
SPECT/CT imaging of mice with SKOV-3 xenografts 48 h after injection of [^177^Lu]Lu-G3 (Left) and [^177^Lu]Lu-G3-ABD (Right). Maximum intensity projection. Full linear scale.

The SPECT/CT image, which was acquired 48 h after an injection of [^177^Lu]Lu-G3 and [^177^Lu]Lu-G3-ABD (Figure 6), was in agreement with the ex vivo measurement data. The uptake of [^177^Lu]Lu-G3 in kidneys and liver was higher than the uptake of [^177^Lu]Lu-G3-ABD. The tumour uptake of [^177^Lu]Lu-G3 was noticeably lower than the uptake of [^177^Lu]Lu-G3-ABD.

To further elucidate the difference between the biodistribution of [^177^Lu]Lu-G3-ABD and [^177^Lu]Lu-ABD-G3, the analysis was additionally performed at 4 and 144 h after injection. A comparison of the results is presented in Table 3. At 4 h after injection, there was no significant difference in the blood concentration of the constructs. However, the clearance rate of [^177^Lu]Lu-ABD-G3 was appreciably higher and its blood half-life (T_1/2_ = 10.8 h) was noticeably shorter than the blood half-life of [^177^Lu]Lu-G3-ABD (T_1/2_ = 19.2 h). The renal uptake of [^177^Lu]Lu-ABD-G3 was already significantly higher than the uptake of [^177^Lu]Lu-G3-ABD at 4 h and remained higher throughout the experiment. [^177^Lu]Lu-G3-ABD had a significantly higher uptake in the liver (approximately two-fold) and spleen (approximately three-fold) starting from 4 h after injection. In addition, there was higher uptake of [^177^Lu]Lu-G3-ABD in the salivary gland, lung, and muscle at 48 h and thereafter. No significant difference in tumour uptake between [^177^Lu]Lu-ABD-G3 and [^177^Lu]Lu-G3-ABD was observed at any time point of the study.

**Figure 7:**
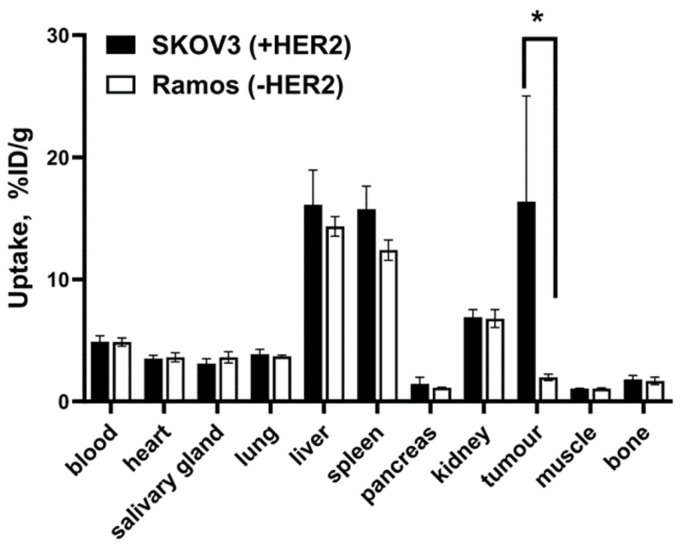
The uptake of [^177^Lu]Lu-G3-ABD in HER2-positive SKOV-3 and HER2-negative Ramos xenografts 48 h after injection. Asterisk (*) marks a significant (*p* < 0.05) difference.

The uptake of [^177^Lu]Lu-G3-ABD in HER2-negative Ramos xenografts was much lower (*p* < 0.05) than the uptake in SKOV-3 xenografts with high HER2 expression (Figure 7). This shows that the in vivo accumulation of [^177^Lu]Lu-G3-ABD in tumours depends on the level of expression of HER2.

**Figure 8:**
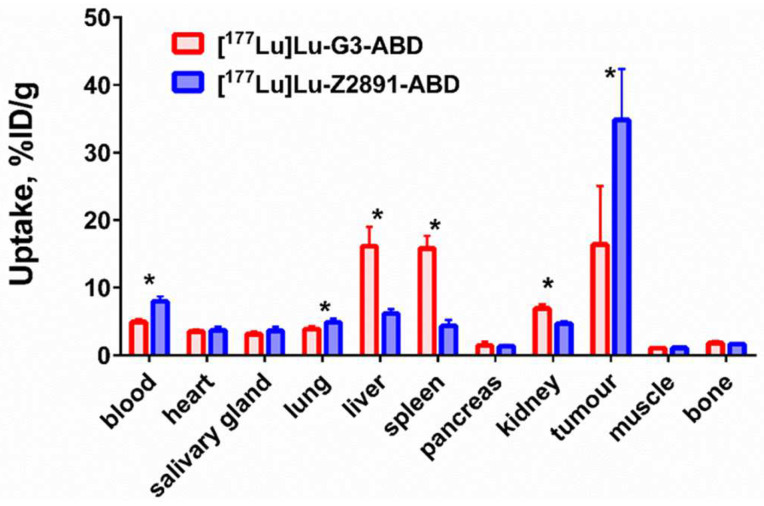
Comparison of the biodistribution of the DARPin G3-ABD and the affibody molecule Z_HER2:2891_-ABD labelled with ^177^Lu in mice bearing SKOV-3 xenografts at 48 h after injection. Asterisk (*) marks a significant (*p* < 0.05) difference.

The comparison of the biodistribution of [^177^Lu]Lu-G3-ABD and the previously studied affibody molecule [^177^Lu]Lu-Z_HER2:2891_-ABD ([^177^Lu]Lu-ABY-027) in HER2-expressing SKOV-3 xenograft-bearing mice 48 h after injection are presented in Figure 8. The blood concentration of [^177^Lu]Lu-G3-ABD was significantly (*p* < 0.0005) lower than for [^177^Lu]Lu-Z_HER2:2891_-ABD (4.9 ± 0.5 and 8.0 ± 0.7%ID/g, respectively). The tumour uptake of [^177^Lu]Lu-Z_HER2:2891_-ABD was two-fold higher than that of [^177^Lu]Lu-G3-ABD (34.8 ± 7.5 and 16.4 ± 8.7%ID/g, respectively). The uptake in liver, spleen, and kidneys was significantly (*p* < 0.0005) higher for [^177^Lu]Lu-G3-ABD (16.1 ± 2.9, 15.8 ± 1.9 and 6.9 ± 0.6%ID/g, respectively) in comparison with those for [^177^Lu]Lu-Z_HER2:2891_-ABD (6.2 ± 0.7, 4.3 ± 0.9 and 4.7 ± 0.4%ID/g, respectively).

## 3. Discussion

The potential of ESPs for radionuclide-based diagnostics has been confirmed by the recent clinical evaluations of several scaffold proteins for imaging using PET and SPECT [12,32]. However, their application for radionuclide therapy necessitates solving the problem of the reabsorption of such proteins in kidneys. Preclinical evaluations have shown that the fusion of an ABD enables successful radionuclide therapy mediated by affibodies or ADAPTs [25,26]. A recent clinical study demonstrated that multiple injections of a fusion of an IL-17A-binding affibody with an ABD are well tolerated by patients [33]. Moreover, biweekly injections for two years did not trigger the formation of neutralising antibodies. Still, scaffold proteins have dissimilar structures and different amino acid compositions. This complicates the translation of information concerning features of molecular design from one scaffold to another because off-target interactions might be very different. Moreover, different scaffold proteins, such as the DARPin G3 and Z_HER2:2891_, bind to different epitopes of HER2 [34,35]. Thus, a potential steric hindrance for binding HER2 due to fusion with the ABD should have a different character for different HER2-targeting proteins. In addition, different scaffold proteins might have unexpected interactions between the targeting domains and the ABD. On the other hand, this opens an opportunity to find the best scaffold type for different targeted applications (molecular imaging, targeted therapy based on blocking of proliferative signalling or delivery of radionuclides, drugs, and toxins) for every particular molecular target. Thus, we included the DARPin G3 in our program for the evaluation of ABD-fused HER2-targeting constructs for radionuclide therapy. Taking into account that the mutual positioning of the HER2-targeting and albumin-binding domains had a clear impact on the retention in blood and renal uptake of affibody-based agents [31], two variants of the fusion architecture were evaluated (Figure 1).

These ABD-fused variants and a non-ABD-fused G3 DARPin were recombinantly produced and coupled with the DOTA chelator, providing conjugates of high purity (Figure S2). An evaluation using SPR demonstrated that both ABD-fused variants were capable of binding to human and murine serum albumin (Table 1). In agreement with previous findings for non-fused ABD_035_ [36], binding to human albumin was stronger than binding to murine albumin. However, the difference between albumin species was not very pronounced in the case of DARPin-fused ABD_035_. Noticeably, the affinity of ABD-G3-DOTA to both human and murine albumins was considerably weaker than the affinity of G3-ABD-DOTA. The most straightforward explanation would be that the fusion with a DARPin domain creates a steric hindrance for the binding of ABD_035_ to albumins. Another possible explanation would be an interaction between amino acids in the binding site of ABD and DARPin. Conceivably, the effect of such hindrance should depend on the mutual positioning of the domains. Still, we considered that an equilibrium dissociation constant of 10^−8^–10^−7^ M should be sufficient for the stable association of both constructs with albumin having a concentration of (5–7) × 10^−4^ M in the blood [27].

The labelling of the DARPins with ^177^Lu was stable, and a simple size-exclusion purification provided high radiochemical purity of the constructs (Figure S4). In vitro studies in the absence of albumin demonstrated that [^177^Lu]Lu-G3-ABD had a preserved specific binding to HER2-expressing cells (Figure 2A) while the specific binding of [^177^Lu]Lu-ABD-G3 was lost (Figure 2B). Surprisingly, the specific binding of [^177^Lu]Lu-ABD-G3 was recovered in the presence of albumin, which was added to mimic the in vivo conditions (Figure 3B). A steric hindrance from the ABD to binding of the DARPin to HER2 would be an unsatisfactory explanation in this case, since binding of [^177^Lu]Lu-ABD-G3 to a bulky albumin should further enhance the hindrance. More likely, an interaction between the DARPin and the ABD in this construct resulted in blocking the paratope of the DARPin in ABD-G3, when the ABD was connected to the N-terminus of the DARPin with a linker that was long enough and flexible. Adding albumin resulted in the preferential binding of the ABD to this protein, releasing the paratope of the DARPin for binding to HER2. This hypothesis is also consistent with the weaker binding of ABD-G3 to both human and murine albumin than the binding of G3-ABD (Table 1).

The LigandTracer measurements of [^177^Lu]Lu-G3-ABD and [^177^Lu]Lu-ABD-G3 binding to living HER2-expressing cells yielded dissociation constants at equilibrium (K_D_) (Table 2), which were in the subnanomolar and low nanomolar range. A 1:2 model indicating a presence of binding sites with higher and lower affinity on HER2, which is expressed on living cells, is common for several proteins targeting HER2 (monoclonal antibodies, DARPins, affibodies, ADAPTs), see e.g., [25,37]. It is most likely associated with conformational changes due to the homo- and heterodimerisation of HER2. Such an affinity should still be sufficient for the efficient association with HER2 in the body.

An internalisation of the targeting proteins after binding to cancer cells is important because it makes tumour uptake irreversible. The internalisation pattern for both [^177^Lu]Lu-G3-ABD and [^177^Lu]Lu-ABD-G3 was similar (Figure 4); thus, the architecture of the fusion protein had no impact on this process. The internalisation was relatively slow, which is typical for both unconjugated DARPin G3 [11] and other HER2-targeting scaffold proteins [25,26]. This is in agreement with a general observation that antibody-based therapeutics are as a rule slowly internalised after binding to malignant cells with high HER2 expression [38]. Overall, none of the tested formats offered an advantage in this aspect in comparison with other ABD-fused scaffold proteins.

The biodistribution data showed that the fusion with ABD increased the blood retention of the DARPins 48 h after injection compared to [^177^Lu]Lu-G3 (Figure 5). The activity concentration of [^177^Lu]Lu-G3-ABD in blood remained 153-fold higher than the concentration of non-fused G3. Furthermore, its uptake in the kidney and liver was reduced 12-fold and 1.7-fold, respectively. The tumour uptake was seven-fold higher than for the non-fused G3, probably due to better bioavailability of G3-ABD in the blood. However, the order of the domains had a strong impact on biodistribution. The blood concentration of [^177^Lu]Lu-ABD-G3 was still much higher than the concentration of [^177^Lu]Lu-G3, but was significantly lower than for [^177^Lu]Lu-G3-ABD. The renal uptake of [^177^Lu]Lu-ABD-G3 was 15-fold higher than that of [^177^Lu]Lu-G3-ABD. The comparison of the biodistribution at different time points (Table 3) showed that [^177^Lu]Lu-ABD-G3 was cleared faster from blood and accumulated in kidneys to a much higher extent.

The binding of [^177^Lu]Lu-ABD-G3 to albumin was an order of magnitude weaker than the binding of [^177^Lu]Lu-G3-ABD. Thus, [^177^Lu]Lu-ABD-G3 is more prone to dissociate from albumin. The non-albumin-bound [^177^Lu]Lu-ABD-G3 was filtered through the glomerular membrane and reabsorbed by the kidneys. The renal blood flow is high (up to 20% of the cardiac output [39]); thus, this effect would be more pronounced with time, while the retention of [^177^Lu]Lu-G3-ABD in blood remained high, and its renal uptake was low.

The uptake in the salivary gland, heart, lung, and muscles followed the blood uptake of the ABD-fused DARPins. However, a higher uptake in the liver and spleen for [^177^Lu]Lu-G3-ABD was already detected 4 h after injection. This suggested that this phenomenon is not associated with higher blood concentration but was caused by a different pattern of off-target interaction of [^177^Lu]Lu-G3-ABD compared to [^177^Lu]Lu-ABD-G3. Overall, [^177^Lu]Lu-G3-ABD provided much higher tumour-to-kidney ratios during this study. Therefore, we considered [^177^Lu]Lu-G3-ABD as the most promising variant for targeted delivery, and it was selected for further characterisation.

A comparison of the biodistribution in mice bearing HER2-positive SKOV-3 and HER2-negative Ramos xenografts showed that the uptake of [^177^Lu]Lu-G3-ABD in tumours was HER2-mediated (Figure 7).

Further, [^177^Lu]Lu-G3-ABD was directly, in the same batch of mice with SKOV-3 xenografts, compared with a homologous affibody-based construct [^177^Lu]Lu-ABY-027 (Figure 8). The affibody-based construct showed a significantly (*p* < 0.05) higher uptake in tumours, but lower uptake in kidneys and liver. Thus, [^177^Lu]Lu-ABY-027 is better suited forradionuclide therapy compared with [^177^Lu]Lu-G3-ABD.

It has to be noted that our experience with other constructs showed that the biodistribution and targeting properties of ABD-fused targeting scaffold proteins depended on the physicochemical properties of the payload [40]. Furthermore, an appreciably higher uptake of target drugs in normal tissues is better tolerated than the uptake of radionuclides [36,40]. It would be worthwhile to evaluate if conjugates of G3-ABD with cytotoxic drugs would be suitable for anti-tumour therapy.

## 4. Materials and Methods

### 4.1. General

A Cyclone Storage Phosphor System and OptiQuant image analysis software (PerkinElmer, Waltham, MA, USA) were used for measuring the radioactivity distribution on instant thin-layer chromatography (iTLC) strips.

In vitro cell studies were performed using HER2-expressing ovarian cancer SKOV-3 and breast cancer BT-474 cells, both obtained from the American Type Culture Collection (ATCC, Manassas, MA, USA). Ramos lymphoma cells (from ATCC) were used to establish HER2-negative xenografts. Cells were cultured in RPMI1640 medium (Sigma-Aldrich, St. Louis, MO, USA), supplemented with 10% for SKOV-3 or 20% for BT-474, of foetal calf serum, 2 mM L-glutamine, 100 IU/mL penicillin, and 100 mg/mL streptomycin.

### 4.2. Production, Purification, and Characterisation of the ABD-Fused DARPin G3 Variants and G3 and Conjugation of the DOTA Chelator

The nucleotide sequence of ABD_035_ was deduced from the previously published amino acid sequence of ABD_035_ [36]. The DARPin G3 gene nucleotide sequence was deduced from the DARPin G3 amino acid sequence deposited in PDB (accession number PDB: 2JAB). The genes were assembled using PCR from chemically synthesised oligonucleotides having partially complementary sequences. The genes for ABD_035_ and G3 were joined together in two combinations (ABD-G3, G3-ABD) using overlap extension PCR [41]. A nucleotide sequence encoding the flexible linker GSSSGSSSGSSSGSSS was inserted between the genes, as well as nucleotide sequences encoding HEHEHE and GEEEC onto the 5′- and 3′-genes termini, correspondingly. All the genes were fused to the 3′-terminus of the SUMO gene using overlap extension PCR [42] and cloned into the pET39b plasmid vector between restriction sites *Nde*I and *Hin*dIII. Expression, isolation, and purification of ABD-G3, G3-ABD, and G3 (see Supplementary Material for resulting amino acid sequences of the constructs) was performed according to the methodology described earlier [42].

The ABD-fused Z_HER2:2891_-ABD affibody molecule was provided by Affibody Therapeutics (Solna, Sweden).

For conjugation of DARPin G3 variants to 1,4,7,10-tetraazacyclododecane-1,4,7-Tris-acetic acid-10-maleimidoethylacetamide (mal-DOTA) (MW = 526.4 mg/mmol), G3-E_3_C (2.650 mg, 0.18 µmol, 500 μL in PBS, pH 7.4) or G3-ABD-E_3_C/ABD-G3-E_3_C (10 mg, 0.04 µmol, 500 μL in PBS, pH 7.4) was incubated with a 280-fold molar excess of dithiothreitol (DTT) (7.8 mg, 50.4 μmol, in 1M solution in PBS, pH 8.0) for 30 min at 40 °C. To remove DTT, the reaction mixture was purified using an NAP-5 column (Cytiva, Uppsala, Sweden), pre-equilibrated with degassed 20 mM ammonium acetate, pH 6.5. The high-molecular-weight fractions containing the proteins were incubated with an 11-fold molar excess of maleimide-DOTA (1 mg, 1.96 µmol, 30 μL in DMSO) at 40 °C for 1 h. To remove the unconjugated chelator, the reaction mixture was purified using a NAP-5 column with PBS elution, pH 8.0. The protein concentration was measured using a DS-11 spectrophotometer (DeNovix, Wilmington, DE, USA). The proteins conjugated to DOTA were stored in PBS (pH 8.0) at −20 °C before labelling with the radiometal. The purity of the constructs was measured using LC-MS as described in [15].

The affinities of the DOTA-conjugated ABD-fused DARPins to human and murine serum albumins were determined using a Biacore T200 instrument (Cytiva, Marlborough, MA, USA) as described by Yin and co-authors [40].

### 4.3. Radiolabelling of DARPins with ^177^Lu and In Vitro Stability

The same labelling protocol was used for the labelling of ABD-fused DARPins (G3-ABD and ABD-G3) and the non-ABD-fused control DARPin G3 with no-carrier-added ^177^Lu (Curium Pharma, Stockholm, Sweden). An aliquot of 40 µg protein in PBS (20 µL, 2 mg/mL) was mixed with 40 µL of 1 M ascorbic acid, pH 6.0, and ^177^Lu (40–200 MBq), and incubated at 60 °C for 1 h. Purification of the radiolabelled DARPins was performed using NAP-5 size-exclusion columns.

Radiochemical yields and radiochemical purities of the ^177^Lu-labeled DARPins were measured using instant thin-layer chromatography (iTLC SG) strips (Agilent, Santa Clara, CA, USA) eluted with 0.2 M citric acid, pH 2.0. The iTLC data were verified using reversed phase-HPLC (see Supplementary Material).

The stability of the ^177^Lu coupling to proteins was tested using a challenge with a 500-fold molar excess of EDTA for 1 h at 37 °C as described earlier [25]. To evaluate the in vitro serum stability, the labelled compounds (0.5 µg of ABD-fused variants and 0.36 µg of G3) were mixed with 100 µg murine serum to mimic a tracer concentration in murine blood immediately after injection. All samples were incubated at 37 °C for 48 h. The stability was analysed using iTLC as described above. The test was performed in duplicates.

### 4.4. In Vitro Studies

The binding specificity of all radiolabelled DARPins to HER2-expressing SKOV-3 and BT-474 cells was evaluated using a saturation experiment performed in triplicate, as described earlier [25]. According to the literature data, SKOV-3 cell express (1.63 ± 0.08) × 10^6^ receptors per cell [43] and BT-474 express (1.3 ± 0.7) × 10^5^ receptors per cell [44]. As an additional control, HER2-negative cell line MDA-MB-468 was used. This cell line expresses 10^3^ receptors per cell [45]. Labelled proteins were added to the cells to obtain the final concentration of 2 nM. HER2 receptors in the control cells were saturated with a 100-fold excess of non-labelled counterpart using 30 min pre-incubation at ambient temperature before adding the radiolabelled protein. After incubation for 1 h at 37 °C, the cells were washed with a cold serum-free medium and detached using a trypsin–EDTA solution. The radioactivity of the cells was measured using an automated gamma counter (2480 Wizard, Wallac, Finland). To evaluate the potential impact of binding of the ABD part to albumin in the blood, these experiments were performed with or without adding human serum albumin. In the case of adding, the albumin concentration during incubation of cells with radiolabelled DARPins was 100 nM.

A LigandTracer Yellow instrument (Ridgeview Instruments, Vänge, Sweden) was used to measure the kinetics (association and dissociation rates) of the ^177^Lu-labelled protein’s interaction with living HER2-expressing SKOV-3 cells as described by Liu and co-workers [25]. The association was recorded with a protein concentration of 3 and 9 nM (1, 3 and 9 nM in the case of [^177^Lu]Lu-G3). The measurements were performed in the presence of human serum albumin (100 nM). The measurements were performed at room temperature to avoid internalisation of bound proteins by the cells. The binding and dissociation data were analysed using the Interaction Map software (Ridgeview Instruments, https://www.ligandtracer.com/product/interaction-map/, last accessed 9 April 2024).

Internalisation of ^177^Lu-labelled ABD-fused DARPins after binding to HER2 was studied using the cell lines SKOV-3 and BT-474. An acid-wash method was applied to distinguish between the internalised and membrane-bound DARPins as described by Liu and co-workers [25]. The internalisation of [^177^Lu]Lu-G3-ABD was evaluated both with and without adding human serum albumin to a concentration of 100 nM. The internalisation of [^177^Lu]Lu-ABD-G3 was evaluated only in the presence of albumin due to the lack of its specific binding to the cells without albumin. The labelled proteins were added to the cells to obtain a final concentration of 2 nM and the cells were incubated at 37 °C. Three culture dishes with cells per time point were used. For efficient removal of membrane-bound proteins, 0.2 M glycine buffer containing 4 M urea, pH 2.0, [25] was used.

### 4.5. In Vivo Studies

The animal experiments were performed in accordance with the national legislation on laboratory animal protection, and the study was approved by the local Ethics Committee for Animal Research in Uppsala (approval number 5.8.18-00473/2021 and date of approval was 26 February 2021). An overdose of Rompun/Ketalar anaesthesia was used for animal euthanasia.

Female BALB/c nu/nu mice with subcutaneously implanted human cancer cells were used for the in vivo studies. SKOV-3 xenografts were used as HER2-positive and Ramos xenografts as HER2-negative models. The average weights of the tumours were 0.26 ± 0.10 g and 0.24 ± 0.14 g, respectively. Four mice per data point were used. The average animal weight was 19.0 ± 1.3 g at the time of the experiment.

The biodistribution of [^177^Lu]Lu-G3-ABD and [^177^Lu]Lu-ABD-G3 in mice with HER2-positive tumours was compared 4, 72, and 144 h after injection as described [25]. The animals were intravenously injected with the conjugates (260 kBq/mouse) in 100 µL of 1% BSA in PBS. The injected protein mass was adjusted to 10 µg/mouse using non-labelled proteins. Additionally, the biodistribution was measured 48 h after injection of an equimolar amount of [^177^Lu]Lu-G3 (7.2 µg in 100 µL of 1% BSA in PBS, 260 kBq/mouse).

To test if the most promising ABD-fused variant, [^177^Lu]Lu-G3-ABD, was accumulated in tumours in a HER2-dependent way, one group of mice with HER2-negative Ramos xenografts was injected with this protein (10 µg in 100 µL of 1% BSA in PBS). The biodistribution was measured at 48 h after injection.

To estimate how the targeting scaffold protein influenced the targeting properties of the ABD-fused constructs, the biodistribution of [^177^Lu]Lu-G3-ABD was compared with the biodistribution of the homologous Affibody-based construct [^177^Lu]Lu-Z_HER2:2891_-ABD (another designation [^177^Lu]Lu-ABY-027). For this purpose, the biodistribution of 33^177^Lu]-ABY-027 was measured 48 h after injection (260 kBq, 5.8 µg in 100 µL of 1% BSA in PBS).

To visualise the biodistribution data, one mouse with a Ramos xenograft and one mouse with a SKOV-3 xenograft were injected with 5 MBq (10 µg) of [^177^Lu]Lu-G3-ABD, and one mouse with a SKOV-3 xenograft was injected with 5 MBq (7.2 µg) of [^177^Lu]Lu-G3. The mice were imaged at 48 h after injection using a nanoSPECT/CT scanner (Mediso Medical Imaging Systems, Budapest, Hungary) as described earlier [25].

## 5. Conclusions

The use of the DARPin G3 for radionuclide therapy is complicated by its high renal reabsorption after clearance via the glomeruli. We tested the hypothesis that a fusion of the DARPin G3 with an ABD would prevent rapid renal excretion and high renal reabsorption, resulting in better tumour targeting. Fusion with the ABD increased the retention time of the anti-HER2 DARPin in blood, resulting in a lower renal uptake and higher accumulation in the tumour due to the higher bioavailability of the targeting agent. The effect of fusion with the ABD depended strongly on the order of the domains in the construct. This is most likely caused by domain–domain interactions. This suggests that different variants of the protein’s architecture should be evaluated in the future development of anti-tumour conjugates based on fusions of engineered scaffold proteins with an ABD.

## Figures and Tables

**Table 1:** Association rate constants (k_a_), dissociation rate constants (k_d_), and equilibrium dissociation constant (K_D_) of DOTA-conjugated ABD-fused G3 derivatives.

	k_a_ (M^−1^s^−1^)	k_d_ (s^−1^)	K_D_ (M)
G3-ABD-DOTA			
Human albumin	1.31 × 10^5^	2.38 × 10^−4^	1.82 × 10^−9^
Murine albumin	6.13 × 10^4^	5.18 × 10^−3^	8.46 × 10^−9^
ABD-G3-DOTA			
Human albumin	8.53 × 10^3^	3.02 × 10^−4^	3.54 × 10^−8^
Murine albumin	2.35 × 10^4^	1.91 × 10^−3^	8.13 × 10^−8^

**Table 2:** InteractionMap evaluation of the affinity of [^177^Lu]Lu-labelled G3 derivatives binding to HER2-expressing SKOV-3 cells in the presence of human serum albumin (100 nM). Weight _1_, % and Weight _2_, %, designate relative abundance of the first and second interaction, respectively.

	K_D1_ (pM)	Weight _1_, %	K_D2_ (nM)	Weight _2_, %
[^177^Lu]Lu-G3-ABD	901 ± 169	54	24 ± 0	45
[^177^Lu]Lu-ABD-G3	1290 ± 920	38	67 ± 14	62
[^177^Lu]Lu-G3	1200 ± 200	60	34 ± 12	40

**Table 3:** Biodistribution of [^177^Lu]Lu-G3-ABD and [^177^Lu]Lu-ABD-G3 in nude mice. Four animals per data point were used.

Uptake, %ID/g						
Organ	[^177^Lu]Lu-G3-ABD			[^177^Lu]Lu-ABD-G3		
	4 h	48 h	144 h	4 h	48 h	144 h
Blood	23.1 ± 2.4	4.9 ± 0.5 ^b^	0.4 ± 0.1 ^c^	21.1 ± 3.4	1.3 ± 0.3 ^b^	0.047 ± 0.004 ^c^
Heart	6.7 ± 0.1	3.5 ± 0.3 ^b^	1.5 ± 0.1 ^c^	6.3 ± 0.8	1.66 ± 0.05 ^b^	0.66 ± 0.03 ^c^
Salivary gland	3.1 ± 0.7	3.1 ± 0.4 ^b^	2.0 ± 0.3 ^c^	2.9 ± 0.2	1.9 ± 0.3 ^b^	0.9 ± 0.1 ^c^
Lung	8.6 ± 1.1	3.9 ± 0.4 ^b^	1.1 ± 0.2 ^c^	8.5 ± 0.9	1.6 ± 0.3 ^b^	0.42 ± 0.02 ^c^
Liver	17.8 ± 1.8 ^a^	16.1 ± 2.9 ^b^	7.3 ± 0.8 ^c^	9.7 ± 0.9^a^	8.6 ± 1.6 ^b^	4.4 ± 0.6 ^c^
Spleen	17.1 ± 3.8 ^a^	15.8 ± 1.9 ^b^	7.0 ± 1.5 ^c^	5.5 ± 1.4^a^	3.7 ± 0.5 ^b^	2.1 ± 0.2 ^c^
Pancreas	1.3 ± 0.1	1.4 ± 0.5	0.6 ± 0.1 ^c^	1.6 ± 0.4	0.8 ± 0.5	0.3 ± 0.1 ^c^
Kidney	7.8 ± 0.6 ^a^	6.9 ± 0.6 ^b^	3.1 ± 0.5 ^c^	39.2 ± 6.0 ^a^	106.4 ± 9.3 ^b^	36.5 ± 6.9 ^c^
Tumour	7.5 ± 5.5	16.4 ± 8.7	7.9 ± 2.5	7.4 ± 5.4	11.3 ± 3.6	4.3 ± 1.7
Muscle	1.0 ± 0.2	1.05 ± 0.04 ^b^	0.48 ± 0.13 ^c^	1.2 ± 0.1	0.51 ± 0.0 ^b^	0.18 ± 0.02 ^c^
Bone	2.1 ± 0.2	1.8 ± 0.3	1.2 ± 0.2	2.4 ± 0.6	1.89 ± 0.04	1.5 ± 0.4
Brain	0.6 ± 0.2	0.17 ± 0.01 ^b^	0.04 ± 0.01 ^c^	0.52 ± 0.18	0.06 ± 0.02 ^b^	0.009 ± 0.003 ^c^

^a^ Significant (*p* < 0.05) difference between [^177^Lu]Lu-G3-ABD and [^177^Lu]Lu-ABD-G3 at 4 h p.i. ^b^ Significant (*p* < 0.05) difference between [^177^Lu]Lu-G3-ABD and [^177^Lu]Lu-ABD-G3 at 48 h p.i. ^c^ Significant (*p* < 0.05) difference between [^177^Lu]Lu-G3-ABD and [^177^Lu]Lu-ABD-G3 at 144 h p.i. *T*-test was used to test for significant differences.

## Data Availability

The data generated during the current study are available from the corresponding author upon reasonable request.

## Supplementary Materials

The following supporting information can be downloaded at: https://www.mdpi.com/article/10.3390/ijms25084246/s1.
