# Supplementary material for: Preclinical Evaluation of HER2-Targeting DARPin G3: Impact of Albumin-Binding Domain (ABD) Fusion
Source: Int J Mol Sci. 2024 Apr 11;25(8):4246. doi: 10.3390/ijms25084246 (PMC11050402; doi:10.3390/ijms25084246)
Supplement: Supplementary file 1 [file ijms-25-04246-s001.zip › ijms-2930241-supplementary.pdf]

Figure S1. Amino acid sequences of the DARPin constructs

The amino acid sequence of G3-ABD was as follows:
MRGSHEHEHEGSDLGKKLLEAARAGQDDEVRILMANGADVNAKDEYGLTPLYLATAHGHLEIVEVLLKNGADVNAVDAIGFTPLHLAAFIGHLEIAEVLLKHGADVNAQDKFGKTAFDISIGNGNEDLAEILQKLNWSSGSSSGSSSLAEAKVLANRELDKYGVSDFYKRLINKAKTVEGVEALKLHILAALPGSEEEC.

The amino acid sequence for ABD-G3 was as follows:
SHEHEHEGSLAEAKVLANRELDKYGVSDFYKRLINKAKTVEGVEALKLHIL-AALPGSSSGSSSGSSSDLGKKLLEAARAGQDDEVRILMANGADVNAKDEYGLTPLYLATAHGHLEIVEVLLKNGADVNAVDAIGFTPLHLAAFIGHLEIAEVLLKHGADVNAQDKFGKTAFDISIGNGNEDLAEILQKLNGEEEC.

The amino acid sequence for G3 was as follows:
MRGSHEHEHEGSDLGKKLLEAARAGQDDEVRILMANGADVNAKDEYGLT-PLYLATAHGHLEIVEVLLKNGADVNAVDAIGFTPLHLAAFIGHLEIAEVLLKHGADVNAQDKFGKTAFDISIGNGNEDLAEILQKLNGEEEC.

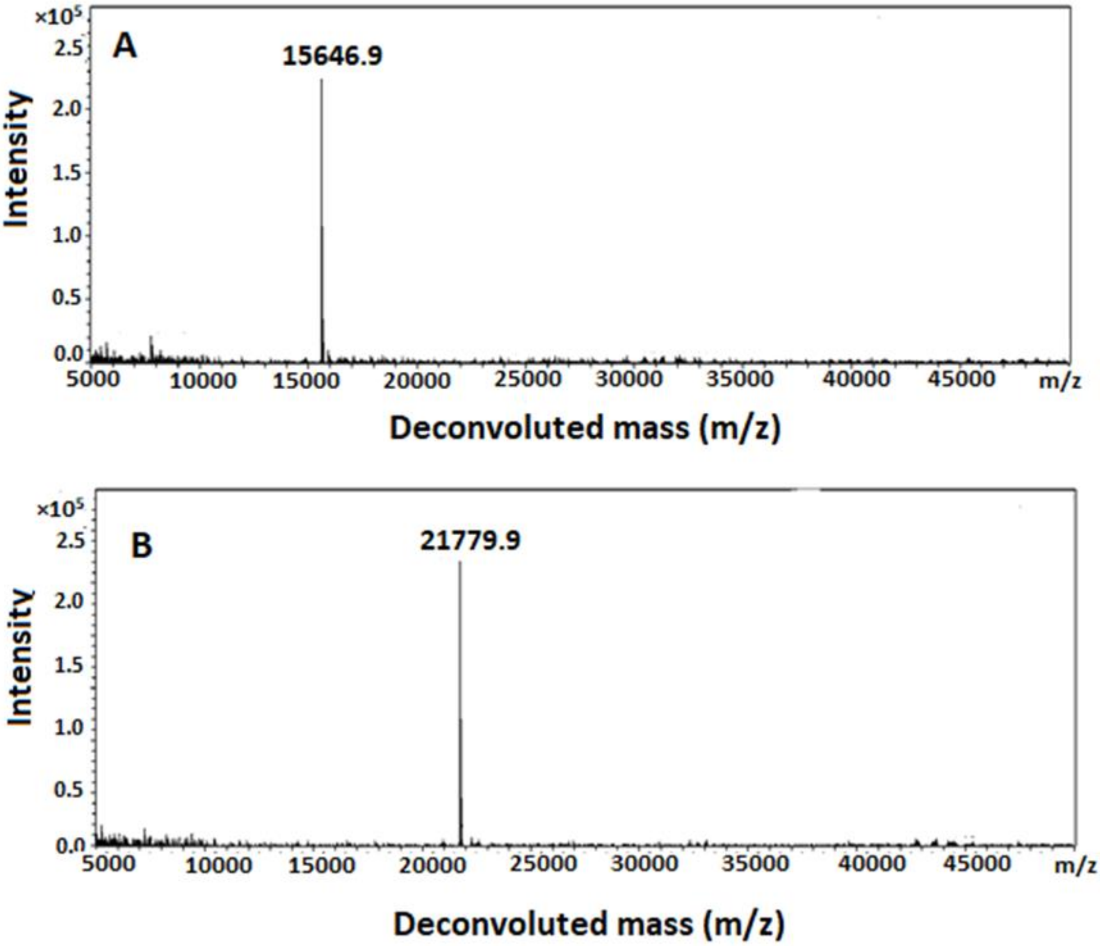

Figure S2. ESI-MS spectra of (A) (G3-DOTA and (B) G3-ABD-DOTA. Calculated molecular weight was 15648.8 and 21779.9 for $(HE)_3$-G3-DOTA and $(HE)_3$-G3-ABD-DOTA. Found molecular weight 15646.9 for G3-DOTA and 21779.9 for G3-ABD-DOTA.

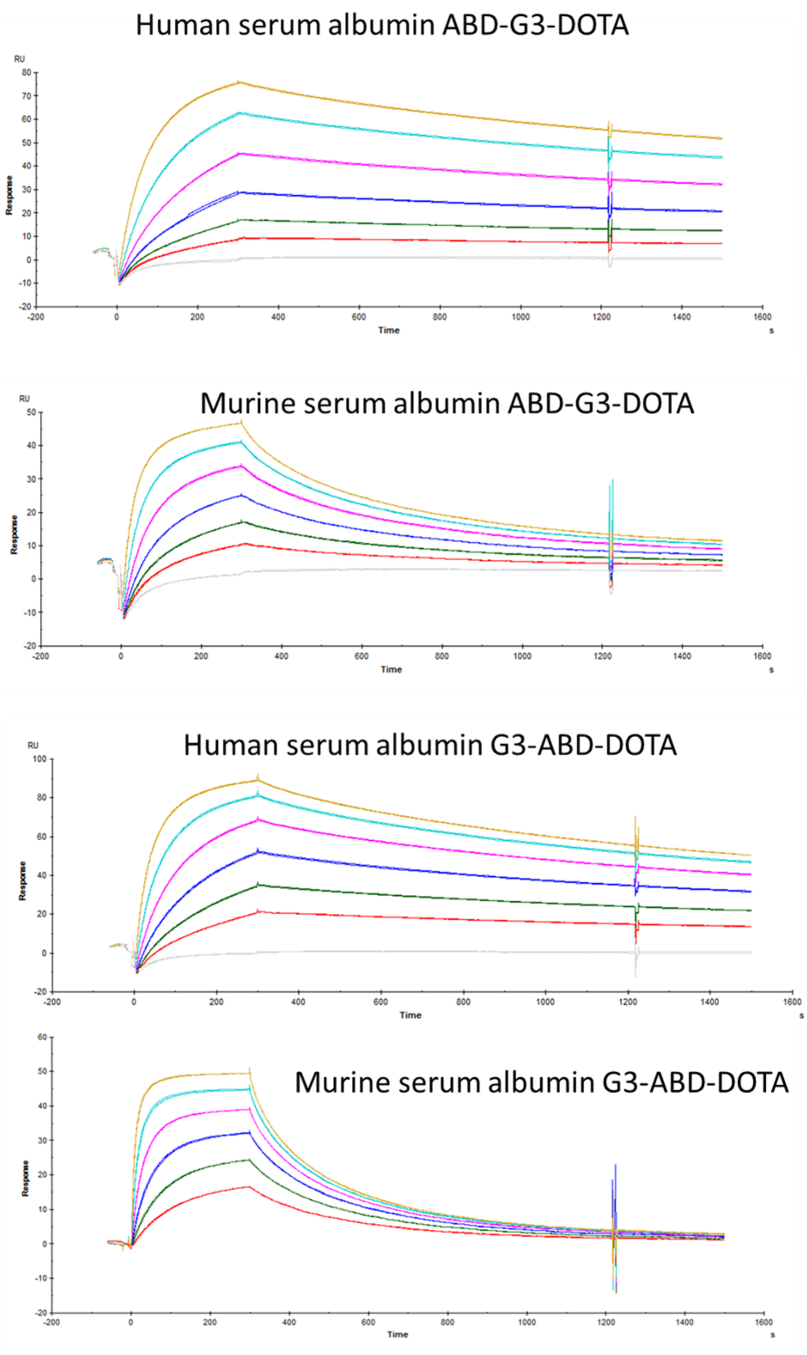

Figure S3. Surface plasmon resonance sensorgrams of ABD-fused DARPins binding to human and murine serum albumin.

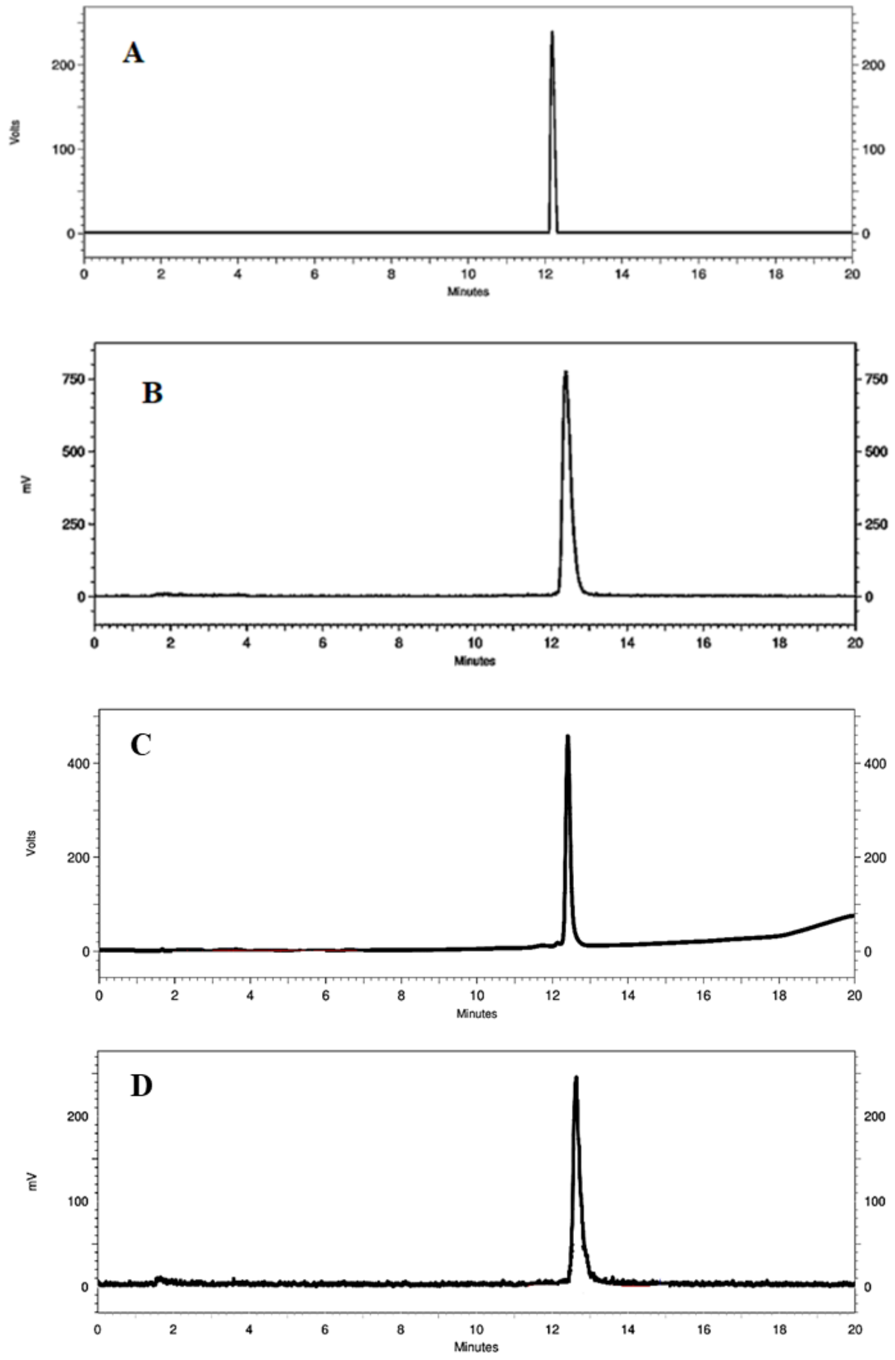

Figure S4. Reversed-phase HPLC chromatograms (UV detection) of non-labelled **(A)** G3-ABD and **(C)** ABD-G3 and radio chromatograms of **(B)** [^{177}Lu]Lu-G3-ABD and **(D)** [^{177}Lu]Lu-ABD-G3.

A Vydac RP C18 column was used for the separation (300 Å; 3 × 150 mm; 5 µm). The solvent A was 10 mM trifluoroacetic acid in water, the solvent B was 10 mM trifluoroacetic acid in acetonitrile. The gradient was 0–15 min from 5% to 70% B, 15–18 min from 70% to 95% B, and 19–20 min at 5% B. The flow rate was 1.0 mL/min. The analysis was performed using the Elite LaChrom system (Hitachi, VWR, Darmstadt, Germany) with a radiation detector (Bioscan, Washington, DC, USA)

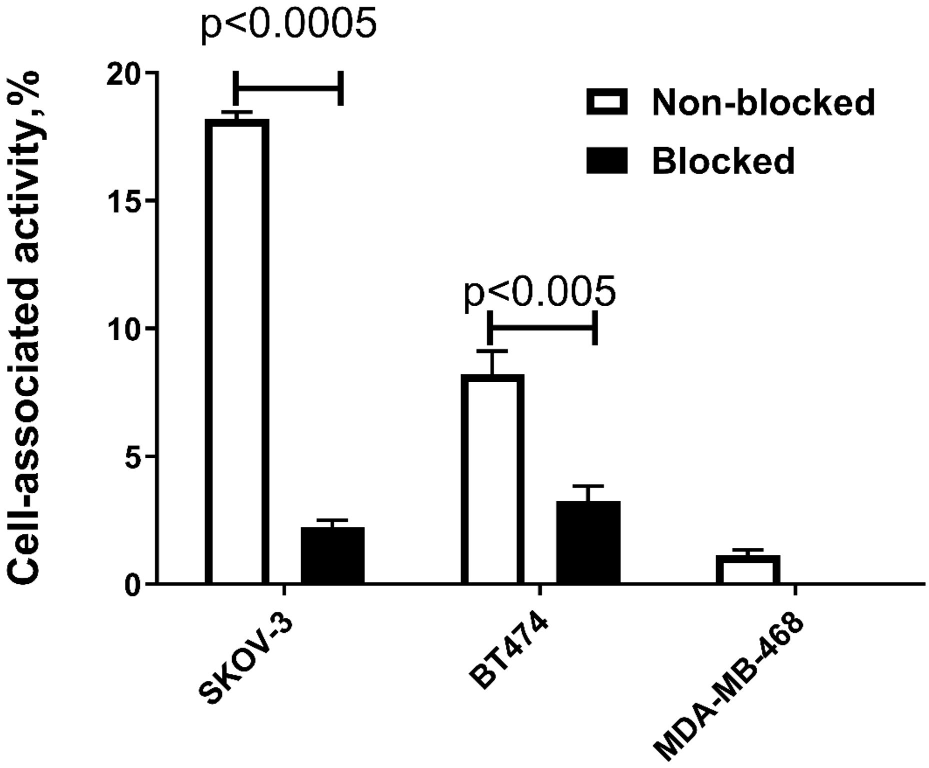

Figure S5. Specificity of [^{177}Lu]Lu-G3 non-ABD-fused control protein binding *in vitro* to the SKOV-3, BT-474 (HER2-positive) and MDA-MB-468 (HER2-negative) cell lines. For the pre-saturation of HER2, a 100-fold molar excess of the non-radioactive DARPin G3 was added before adding the labelled conjugate (2 nM). The data are presented as an average value from three samples ± SD.
